# The *SAMHD1* rs6029941 (A/G) Polymorphism Seems to Influence the HTLV-1 Proviral Load and IFN-Alpha Levels

**DOI:** 10.3389/fcimb.2020.00246

**Published:** 2020-05-25

**Authors:** Maria Alice Freitas Queiroz, Ednelza da Silva Graça Amoras, Tuane Carolina Ferreira Moura, Carlos Araújo da Costa, Maisa Silva de Sousa, Sandra Souza Lima, Ricardo Ishak, Antonio Carlos Rosário Vallinoto

**Affiliations:** ^1^Laboratory of Virology, Institute of Biological Sciences, Federal University of Pará, Belém, Brazil; ^2^Laboratory of Cellular and Molecular Biology, Tropical Medicine Center, Federal University of Pará, Belém, Brazil

**Keywords:** HTLV-1, SAMHD1, polymorphism, IFN-α, symptomatic

## Abstract

SAMHD1, a host dNTPase, acts as a retroviral restriction factor by degrading the pool of nucleotides available for the initial reverse transcription of retroviruses, including HTLV-1. Polymorphisms in the *SAMDH1* gene may alter the enzymatic expression and influence the course of infection by the virus. The present study investigated the effect of polymorphisms on HTLV-1 infection susceptibility and on progression to disease in 108 individuals infected by HTLV-1 (47 symptomatic and 61 asymptomatic) and 100 individuals in a control group. *SAMHD1* rs6029941 (G/A) genotyping and HTLV-1 proviral load measurements were performed using real-time PCR and plasma IFN-α was measured by ELISA. Polymorphism frequency was not associated with HTLV-1 infection susceptibility or with the presence of symptoms. The proviral load was significantly higher in symptomatic individuals with the G allele (*p* = 0.0143), which presented lower levels of IFN-α (*p* = 0.0383). *SAMHD1* polymorphism is associated with increased proviral load and reduced levels of IFN-α in symptomatic patients, and may be a factor that contributes to the appearance of disease symptoms.

## Introduction

HTLV-1 is responsible for the development of HTLV-1-associated myelopathy/tropical spastic paraparesis (HAM/TSP) and adult T-cell leukemia/lymphoma (ATLL) and is associated with other inflammatory syndromes, such as rheumatoid arthritis, dermatitis, and uveitis, in addition to autoimmune diseases (Quaresma et al., 2015). However, most infected individuals do not develop symptoms, and parameters for evaluating the clinical outcome of each carrier remain undefined (Bangham et al., 2015). Therefore, several studies have investigated factors, mainly genetic factors that can elucidate the course of HTLV-1 infection in the onset of infection-related symptoms (Talledo et al., 2010; Assone et al., 2018; Vallinoto et al., 2019).

SAMHD1 is a deoxynucleotide triphosphate triphosphohydrolase (dNTPase) that acts as an intrinsic factor of retroviral restriction, degrading the pool of nucleotides available for the initial reverse transcription, limiting the replication of retroviruses, including HTLV-1 (van Montfoort et al., 2014). Blocking this step prevents the synthesis of double-stranded DNA and disrupts the later stages of the viral replication cycle, including nuclear translocation and integration of DNA into the genome of the host cell (Sze et al., 2013b).

Genetic variations in the *SAMDH1* gene may alter the expression of the enzyme and influence the course of viral infection. A polymorphism in the *SAMHD1* 3′-UTR region, rs6029941 (A/G), seems to alter enzyme expression, where the A allele is associated with higher levels of *SAMHD1* expression and the G polymorphic allele is associated with lower levels (Zhu et al., 2018). In this regard, individuals infected by HTLV-1 with reduced SAMHD1 levels may have a greater proviral load, whereas increased enzyme expression may reduce viral replication and activate a potent type I IFN response, which would enable infection control (van Montfoort et al., 2014). The aim of the present study was to evaluate the effect of the *SAMHD1* polymorphism rs6029941 (A/G) on the proviral load and the development of symptoms of HTLV-1-associated diseases.

## Materials and Methods

### Study Population and Sample Collection

The present study included blood samples from 108 individuals infected with HTLV-1 (22 clinically diagnosed with HAM/TSP, 18 with rheumatic manifestations, 3 with dermatitis, 1 with uveitis, 3 with more than one diagnosis and 61 asymptomatic) treated at the Tropical Medicine Center outpatient clinic of the Federal University of Pará. The patients were of both sexes, were older than 18 years of age and had not been treated with glucocorticoids. The control group included 100 individuals at risk of infection but not infected with the HTLV-1/2, HIV-1, hepatitis B or C, *Chlamydia trachomatis* or syphilis viruses, to compare polymorphism frequencies.

A 10 mL blood sample was collected by intravenous puncture using a vacutainer system containing ethylenediaminetetraacetic acid as an anticoagulant. The samples were centrifuged and separated into plasma and a leukocyte mass. The leukocyte samples were used to extract genomic DNA for analysis of the SAMHD1 rs6029941 (A/G) polymorphism and quantification of the proviral load.

### DNA Extraction

DNA was extracted from peripheral blood leukocytes using the Puregene kit (Gentra Systems, Minneapolis, MN, USA) according to the manufacturer's protocol, which included cell lysis, protein precipitation, and DNA precipitation and rehydration. DNA was quantified using a Qubit® 2.0 fluorometer (Life Technologies, Carlsbad, CA, USA) and Qubit™ DNA assay kit reagents (Life Technologies, Carlsbad, CA, USA), following the protocol recommended by the manufacturer.

### Quantification of HTLV-1 Proviral Load

Proviral load was quantified using a quantitative real-time PCR using three target sequences, synthesized through the TaqMan® system (Life Technologies, Foster City, CA, USA), according to a previously described protocol (Tamegão-Lopes et al., 2006). Samples containing 5 mL of whole blood were collected for leukocyte DNA extraction, followed by relative quantification using real-time PCR. The results were subsequently adjusted for the absolute proviral quantity, based on leukocyte counts per μL, and expressed as proviral DNA copies/μL.

### Genotyping of *SAMHD*1 rs6029941 (A/G)

The polymorphism, located in the UTR3′ region of the gene, was genotyped by real-time PCR using a StepOnePLUS™Real-Time PCR System. The reaction consisted of a commercial assay (C__29973868_10) containing primers and specific TaqMan® probes for amplification of the target sequence (Thermo Fisher, Carlsbad, California, USA). The reaction contained 1× MasterMix, H_2_O, 20× C_11537906_20 assay buffer and 50 ng of DNA, which was subjected to the following cycling conditions: 10 min at 95°C and 40 cycles of 15 s at 95°C and 1 min at 60°C.

### Quantification of Plasma IFN-α

Plasma IFN-α was measured by the enzyme-linked immunosorbent assay (ELISA) Invitrogen Human IFN alpha ELISA Kit (ThermoFisher, Carlsbad, CA, USA), which uses specific monoclonal antibodies to detect the cytokine and followed the manufacturer's instructions.

### Statistical Analysis

The genotype frequencies were estimated by direct count. The allele frequency was calculated using the formula: F = 2 × number of homozygous individuals + number of heterozygous individuals/total number of individuals. The sum of the two alleles must equal 1. This is the standard form of scientific literature in the field of genetics to describe allele frequencies (the allele frequency described in the table not corresponding to “*n*” and %).

Differences between genotype frequencies observed in the investigated groups were calculated by the χ^2^ (chi-square) test. The proviral load and plasma IFN-α were compared between groups using the non-parametric Kruskal-Wallis and Mann-Whitney test. All tests were performed using BioEstat 5.3 software. Associations with *p* < 0.05 were considered statistically significant.

## Results

The distributions of the allele and genotype frequencies of the rs6029941 (A>G) polymorphism were similar between individuals infected with HTLV-1 and the control group, with a higher frequency of the polymorphic allele (SAMDH1^*^G) in individuals with the virus, but without statistical significance (Table 1). Among the infected individuals, no statistically significant difference was observed between the asymptomatic group and the patients with different symptom manifestations (including patients with HAM/TSP, rheumatic manifestations, dermatitis, and uveitis) (Table 1).

**Table 1:** Genotype and allele frequencies of *SAMHD1* rs6029941 (A>G) polymorphism among HTLV-1 carriers and in the control group and among asymptomatic and symptomatic HTLV-1 carriers.

**Genotypes and alleles**	**HTLV-1**	**Control**	p^**^	**Asymptomatic**	**Symptomatic**	p^**^
	***n* = 108**	***n* = 100**		***n* = 61**	***n* = 47**	
	***n* (%)**	***n* (%)**		***n* (%)**	***n* (%)**	
AA	48 (33.3)	52 (52.0)	0.3339	25 (41.0)	23 (48.9)	0.5236
AG	41 (50.0)	37 (40.0)		26 (42.6)	15 (31.9)	
GG	19 (16.7)	11 (11.0)		10 (16.4)	09 (19.2)	
^*^A	0.63	0.71	0.2925	0.62	0.64	0.8836
^*^G	0.37	0.29		0.38	0.36	

*n, number of individuals*.

** *Chi-square test*.

* *allele*.

The proviral load test was performed only on 47 samples and the plasma measurement of IFN-α in 52 samples from individuals infected with HTLV-1, because not all samples were viable for these tests.

**Figure 1:**
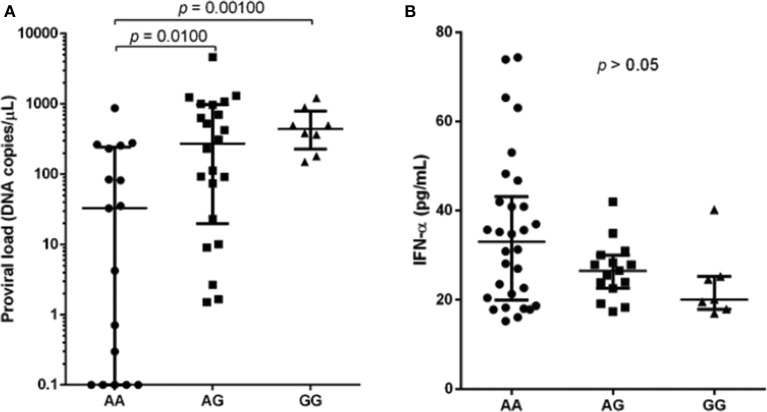
Proviral load **(A)** and IFN-α levels **(B)** among HTLV-1 infected individuals with different genotypes for the SAMHD1 rs6029941 (A > G) polymorphism. Kruskal-Wallis test.

The median of proviral load was higher in individuals infected carrying the polymorphic allele (AA: 33.95, AG: 270.1 and GG: 424.2), and significant difference was observed between wild-type and polymorphic genotypes, AG and GG (Figure 1A; *p* = 0.0100 and *p* = 0.0010, respectively). In contrast, median IFN-α levels were lower in individuals with polymorphic genotypes (AA: 33.04, AG: 26.52 and GG: 20.10) but without statistical significance (Figure 1B; *p* = 0.1246).

**Figure 2:**
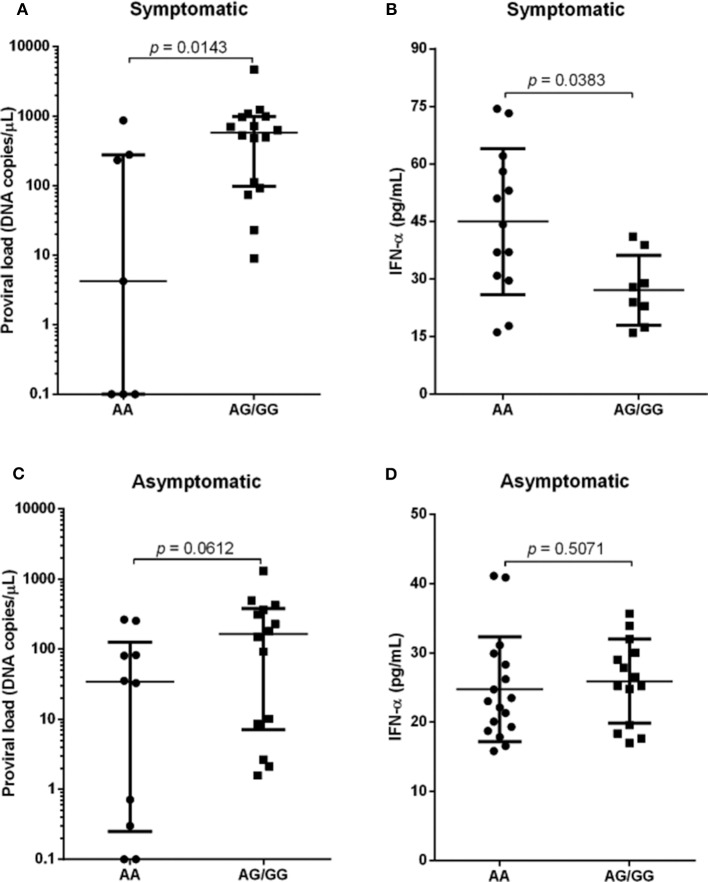
Proviral load and IFN-α levels among individuals with different genotypes for the SAMHD1 rs6029941 (A>G) polymorphism according to the presence **(A,B)** and absence of symptoms **(C,D)**. Mann-Whitney test.

Analyzes of proviral load and IFN-alpha levels were performed among individuals with wild genotype (AA), related to greater expression of SAMHD1, compared to individuals with genotypes expressing the polymorphic allele (^*^G) in homo and heterozygosis (AG and GG), which are associated with reduced expression of the restriction factor. The viral load was significantly higher in symptomatic individuals with polymorphic genotypes, *p* = 0.0143 (Figure 2A), who had lower levels of IFN-α, *p* = 0.0383 (Figure 2B). Analysis of the asymptomatic group showed higher median levels of proviral load in individuals with polymorphic genotypes, although it is not statistically significant (Figure 2C). There was no difference in IFN-α levels (Figure 2D).

## Discussion

Restriction factors are important components of innate immunity that recognize specific patterns of retroviruses and inhibit viral replication. The main restriction factors associated with the inhibition of retroviruses include APOBEC3, TRIM5α, Tetherin, and SAMHD1 (Wilkins and Gale, 2010). The SAMHD1 enzyme restricts infection by degrading the pool of nucleotides available for viral reverse transcription. Furthermore, SAMHD1 undergoes specific conformational changes that promote signaling for the production of type I interferon and the expression of proinflammatory cytokines by the infected cell (van Montfoort et al., 2014).

In the present study, the frequency of the *SAMHD1* rs6029941 (A/G) polymorphism was not associated with infection susceptibility or with the presence of HTLV-1-related symptoms. These results may be related to the small sample size used in the study. Although the Amazon region is endemic for HTLV-2 infection, found mainly in the indigenous population (Ishak et al., 2003; Braço et al., 2019), the prevalence of HTLV-1 is low, approximately 0.9% among blood donors (Catalan-Soares et al., 2005). However, the sample size of the study corresponds to the number of patients who are attending the outpatient clinic at the Center for Tropical Medicine at the Federal University of Pará, a place that monitors patients diagnosed with HTLV-1 in the city of Belém.

Another possibility of the lack of association of the frequency of *SAMHD1* rs6029941 (A/G) polymorphism with the symptoms of the diseases is that it may not be associated with the development of symptoms of all types of diseases associated with HTLV-1, because, although they are of inflammatory etiology, they activate different immunological mechanisms (Quaresma et al., 2015). Thus, these data show that frequency analysis alone is not sufficient to determine the influence of polymorphism on the development of HTLV-1 infection. To better assess this relationship, the levels of proviral load and IFN-α were analyzed, and the results showed that polymorphism could act as a possible factor that would contribute to the complex manifestations of the symptoms of the disease.

There was an association between the *SAMHD1* rs6029941 (A/G) polymorphism and variations in the HTLV-1 proviral load. The GG polymorphic genotype, related to lower enzyme levels (Zhu et al., 2018), was associated with a higher proviral load in individuals infected with HTLV-1, regardless of the presence or absence of infection-related symptoms. These results corroborate recent data indicating that this polymorphism reduces SAMHD1 gene expression (Zhu et al., 2018) because reduced SAMHD1 levels favor HTLV-1 replication, which results in an increased proviral load.

The AA genotype, which conferred greater expression of *SAMHD1*, was associated with lower levels of proviral load, which may be related to better control of HTLV-1 replication. Higher levels of SAMHD1 would restrict infection by degradation of the nucleotides pool for reverse transcription. Although it has been demonstrated that HTLV does not appear to be affected by SAMHD1, this finding could be related to a possible escape mechanism of the virus to the restriction factor (Gramberg et al., 2013). However, Sze et al. (2013a) observed that SAMHD1 inhibited reverse transcription in monocytes infected with HTLV-1, leading to the formation of reverse transcription intermediates, responsible for inducing apoptosis and limiting infection. Although the present study did not evaluate a specific type of cells, the results suggest that the polymorphism could favor escape mechanisms of the virus against the control of the innate immune system, influencing the evolution of the infection.

Mutations in the *SAMHD1* gene may alter enzyme synthesis and result in uncontrolled inflammatory responses, mainly mediated through the increased production of type I IFN (Rice et al., 2009). Mutations in the *SAMHD1* gene are associated with an autoimmune disorder through the irregular response of type I IFN, which characterizes Aicardi-Goutières syndrome, in which there is marked production of IL-12 and TNF-α (White et al., 2017).

An important aspect that needs to be considered is the genetic background of the population analyzed in this study, which results from interethnic crossbreeding of Europeans, Indians, and Africans (Santos et al., 2009). Therefore, these preliminary data seem to suggest that the *SAMHD1* rs6029941 (A/G) polymorphism may influence HTLV-1 infection in the evaluated tri-hybrid population. However, because this is the first study that investigated the association of polymorphism in HTLV-1 infection, it will also be necessary to analyze its relationship in other different infected ethnic groups to determine its relevance in other populations.

The choice of the SAMHD1 rs6029941 polymorphism (A/G) was based on its influence on changes in gene expression and because it has not yet been evaluated for HIV and HTLV infection. Although the frequency of polymorphism is not associated with the presence of disease symptoms, it was associated with a higher proviral burden in symptomatic patients, and patients without symptoms, also had higher levels. Possibly, the polymorphism, related to the lower expression of SAMHD1, could promote less inhibition of reverse transcription, leading to the formation of few reverse transcription intermediates (RTIs) and low type I interferon production, resulting in a more productive infection, with a high proviral load (van Montfoort et al., 2014).

The findings found in the present study suggest that only *SAMHD1* rs6029941 (A/G) polymorphism is not able to induce the progression and worsening of the infection, but it would act as a factor that could increase the proviral load and contribute to the appearance of symptoms. Other studies, including the follow-up of asymptomatic patients with polymorphism, may better clarify its influence on the development of symptoms associated with HTLV-1.

In summary, the results suggest that the *SAMHD1* rs6029941 (A/G) polymorphism is associated with increased HTLV-1 proviral load and lower levels of IFN-α in symptomatic patients. Thus, the polymorphism could contribute to the development of the symptoms of the disease.

## Data Availability Statement

The datasets used and analyzed during the current study are available from the corresponding author on reasonable requests from a qualified researcher.

## Ethics Statement

The studies involving human participants were reviewed and approved by the Research Ethics Committee of the Health Science Institute of the Federal University of Pará (protocol no. 2872434/2018). The patients/participants provided their written informed consent to participate in this study.

## Author Contributions

MQ and EA designed the study. TM, CC, and MS provided technical assistance and executed the experiments. MQ, EA, SL, and AV analyzed and interpreted the date. MQ, RI, and AV wrote the manuscript with input from all authors. All authors read and approved the final manuscript.

## Conflict of Interest

The authors declare that the research was conducted in the absence of any commercial or financial relationships that could be construed as a potential conflict of interest.
